# Measuring the impact of rare diseases in Tasmania, Australia

**DOI:** 10.1186/s13023-024-03343-2

**Published:** 2024-10-28

**Authors:** Philippa Scanlon, Garry Ridler, Genevieve Say, Miranda Kellett, Jac Charlesworth, Amanda Neil, Joanne L. Dickinson, Kathryn Burdon, Matthew Jose, Mathew Wallis

**Affiliations:** 1 Tasmanian Clinical Genetics Service, Tasmanian Health Service, Hobart, Australia; 2 Menzies Institute for Medical Research, University of Tasmania, Hobart, Australia; 3 School of Medicine, University of Tasmania, Hobart, Australia; 4 Department Nephrology, Tasmanian Health Service, Hobart, Australia; 5 Department of Health, Health Information Communication Technology, Hobart, Australia

**Keywords:** Rare diseases, Data linkage analysis, Inpatient admissions, Costs

## Abstract

**Background:**

An ongoing challenge with rare diseases is limited data and, consequently, limited knowledge about the collective prevalence and impact of these conditions on individuals, families, and the health system, particularly in rural and regional areas. Using existing datasets, this project aimed to examine the epidemiology of and hospital activity for Tasmanians with rare diseases.

**Methods:**

Rare diseases were defined as non-infectious diseases with a prevalence of less than 1 in 2000. An initial resource set of 1028 ICD-10-AM diagnostic codes was used to identify a cohort of Tasmanians with rare diseases in Tasmanian Health datasets (1 January 2007 until 31 December 2020). Validating the resource set using a small group with known rare diseases revealed limitations in ascertainment, and so an expanded set of 1940 ICD-10-AM diagnostic codes was developed by cross-referencing ICD-10-AM codes with Orphanet data. Cohort hospital activity and admission costs were compared to statewide data for the final year of the study, 01 January 2020 to 31 December 2020.

**Results:**

Using the resource set of 1028 ICD-10-AM diagnostic codes, the period prevalence of rare diseases in Tasmania across all age groups was estimated at 3.5%, with a point prevalence of 1.5% in December 2020. In 2020, 3384 individuals within the Tasmanian rare disease cohort, representing 0.6% of the Tasmanian population, accessed the public hospital system and accounted for 5.6% of all admissions. The mean length of stay for rare disease-related hospital admissions was 5.0 days, compared to 3.3 days for non-rare disease-related admissions. The mean cost per admission for the rare disease cohort was AUD$11,310, compared to AUD$6475 for all admissions statewide. In 2020, using the expanded resource set, the total cost of public hospital admissions in Tasmania was estimated to be AUD$979 million, with rare disease-related hospital admissions accounting for 9.1% of this cost, increasing to 19.0% when the costs for all admissions for the rare disease patients were included.

**Conclusions:**

Patients with rare diseases had more admissions, longer length of stay, and a higher average cost per admission. Patients with rare diseases have a disproportionate impact on statewide hospital activity and costs in Tasmania.

**Supplementary Information:**

The online version contains supplementary material available at 10.1186/s13023-024-03343-2.

## Background

Rare diseases (RDs) are typically defined as conditions affecting fewer than one person per 2000 individuals in the population [1]. There are over 7000 known RDs, which are primarily genetic in aetiology, and more than half affect children [2]. While individually rare, RDs collectively impact a significant portion of the Australian population [3–6]. Excluding rare cancers, infectious diseases and poisonings with an acute or subacute clinical course, the point prevalence of RDs has been conservatively estimated to affect 3.5–5.9% of the worldwide population [7].

RDs have gained recognition as a global public health priority due to their profound impact on affected individuals and communities worldwide [8]. Due to the associated workforce and health care needs, the Australian Government Department of Health commissioned an Action Plan, which acknowledged the need to measure the collective impact of RDs on the community [9].

Ensuring comprehensive, accurate and unambiguous diagnostic codes is vital to facilitate RDs recognition and, ultimately, timely access to appropriate care for patients. While hospital activity data can provide a mechanism to identify RDs [10], there are significant challenges pertaining to accurate assessment [11]. Most notably, this is because RDs are underrepresented in established health data collection systems, such as the tenth revision of the International Statistical Classification of Diseases (ICD-10) [12]. In response to the underrepresentation of RDs in the ICD-10, the World Health Organization established a Topic Advisory Group for RDs, managed by the international consortium, Orphanet. The aim was to help establish the eleventh revision of the International Statistical Classification of Diseases (ICD-11), which now encompasses nearly 5500 RDs [12]. Orphanet formulated a classification and coding system for RDs, known as ORPHAcodes, and they maintain an inventory of RDs with unique ORPHAcodes cross-referenced with both ICD-10 and ICD-11 [11]. ORPHAcodes identify clinically unique and distinct entities with prevalence equal to no more than 1 in 2000 in the general European population. In Australia, the dominant health coding system is an Australian Modification of ICD-10 (ICD-10-AM) [4], with the ICD-11 anticipated to be adopted in around 2031, following the commitment established during the 2019 World Health Assembly meeting [13].

Understanding the impact of RDs in Tasmania is important because research studies conducted globally, including in Western Australia, Hong Kong, Ireland, and Italy, have consistently highlighted the substantial economic burden of RDs [4, 14–16]. In the United States, it was estimated that RDs incurred an annual cost of approximately USD$968 billion in 2019, encompassing direct medical expenses, indirect costs related to reduced productivity, non-medical expenses, and healthcare services not covered by insurance [17]. Tasmania is a small island state with a dispersed but stable populace exceeding 540,000 individuals. Despite recent progress, Tasmania still lags other Australian states and territories in several health measures, largely related to its geography and socioeconomic disparities [18, 19]. Understanding the prevalence of RDs at both individual and collective levels is crucial for designing efficient pathways for diagnosis and management, and location-specific data can enable pathways to be tailored to ensure equitable service provision. The main aim of this retrospective cohort study was to determine the prevalence of RDs in Tasmania and assess whether RDs result in a significant and disproportionate impact on Tasmanian hospital activity and costs. A secondary aim of the study was to develop an improved Rare Diseases ICD-10-AM resource set.

## Methods

For the purposes of this study, a rare disease (RD) was defined as any non-infectious disease with a prevalence of less than 1 in 2000 in the residential Tasmanian population.

### Initial cohort identification

Using the 1084 ICD-10-AM RD codes made available by Walker et al. [4], an initial RD cohort was identified from the following Tasmanian Health datasets: Tasmanian Public Hospital Admitted Patient Episodes (01/01/2007–31/12/2020); Tasmanian Public Hospital Emergency Department Presentations (01/01/2000–01/12/2020); Tasmanian Cause of Death/Fact of Death (01/01/2006–31/12/019) (Additional file 1—Linkage Diagram). A control cohort was established by creating a matched comparator group with no RDs, i.e., lacked any of the 1084 ICD-10-AM codes in any diagnosis field, and were of the same age, gender, and geographical location (SA2).

### Data extraction and linkage

Linkage between these datasets was managed and undertaken by the Tasmanian Data Linkage Unit (TDLU), which has well-established and published data linkage processes [20]. Datasets were linked by a Project Person Identifier (PPID), with the same PPID representing an individual in the population. In accordance with the ‘Separation Principle’ used by the TDLU, no individual identifying information was released to the researchers. Study variables (Additional file 2—Study Variables, Table 1) for all available inpatient hospital records and Emergency Department presentations were provided to the researchers by data custodians. Researchers assigned corresponding ORPHAcodes and Orphanet linearisation parents to the data provided. The Tasmanian Cause of Death/Fact of Death database was used to identify those in the cohort who had died, along with the cause of death.

**Table 1:** Comparison of patient characteristics of the RD and control cohorts in the linked dataset

	Number of ICD-10-AM RD codes (1028) present in dataset	Number of individuals across study period	% Male^2^	Median age (years) (as at 31/12/2020 or death)^2^	Number of deaths (%)	Median age at death (years)	Number alive Dec 2020	% Tas Population Dec 2020 (565,557)
RD cohort	663 (64.5%)	13,806	52.0	61	5151 (37.3)^1^	72	8655	1.5
Control cohort	0	12,545	52.1	61	2370 (18.9)	77	10,175	1.8

^1^The cause of death was RD-related in half of all deaths in the RD Cohort (2589, 50.3%)

^2^The control cohort was age, sex and geographical location matched

To overcome potential differences in the Tasmanian population over the data collection period, we estimated the period prevalence for each ICD-10-AM RD code in the researchable datasets provided by the data custodians. The period prevalence for each RD was assessed as the number of people who were admitted to hospital for that disease during the study period, divided by the Tasmanian population size at the midpoint of the period (December 2013) [21]. A total of 11 diseases (44 ICD-10-AM codes) were determined to not be rare in the Tasmanian population and were removed (Additional file 3—Removed Codes) to establish the ‘Stringent Rare Diseases Resource Set’ of 1028 ICD-10-AM codes cross-referenced to 457 ORPHAcodes. This resource set is provided in Additional file 4—Stringent Rare Diseases Resource Set.

The main RD cohort for analysis included all individuals who had a record in either of the linked Tasmanian Health datasets with one of the 1028 ICD-10-AM codes from the Stringent Resource Set recorded in any diagnosis field or cause of death field; and a hospital admission date, Emergency Department presentation date or death date between 1 January 2007 and 31 December 2020. Statistics describing the frequency and characteristics of patients and admissions were calculated for the study period. Using the linked data, hospital admissions and Emergency Department presentations were examined for the entire study period (1 January 2007 to 31 December 2020). Lengths of stay (LOS) were calculated as the difference between the discharge date and time and the admission date and time, rounded to the nearest integer.

### Developing an expanded resource set

To validate the ability to identify RD patients in datasets based on the Stringent Resource Set, a list of 300 patients with a known RD diagnosis, along with the corresponding ICD-10-AM and ORPHAcode, was provided to a data separator. The data separator randomly selected 100 individuals from that list and provided TDLU with the linkage variables for those 100 individuals only. This cohort is referred to as the validation cohort, and TDLU used these linkage variables to identify PPIDs for the cohort. The data separator then provided researchers with 100 PPIDs with their corresponding known RD diagnosis. We then reviewed all admissions and Emergency Department presentations for the validation cohort to identify if they were captured by ICD-10-AM coding in any diagnosis field. This validation process led to embarking on the development of an expanded ICD-10-AM RDs resource set, to compare its performance to the Stringent Resource Set.

In developing an expanded resource set, referred to as the ‘Tasmanian Rare Diseases Resource Set’, a list of all available ICD-10-AM codes (12th edition) cross-referenced to ICD-10 codes was obtained for review, noting there are seven different ICD-10-AM editions over the study period, and supplementary codes for chronic conditions were introduced in 2015 [22, 23]. The ‘Chronicle’ resource on the Independent Health and Aged Care Pricing Authority (IHACPA) website describes changes from ICD-10-AM first edition to twelfth edition [24]. This ICD-10-AM list was then cross referenced to alignment data available through the Orphanet Knowledge Base [25], as well as a list of rare cancers [26] developed through the European RARECARE project [27, 28], and the Walker et al. [4] list. The aligned list included linearisation data, and enabled filtering by Orphanet classification with the ability to visualise all ORPHAcodes, and thus ICD-10/ICD-10-AM codes, under that class. Orphanet provides alignment data to indicate if the cross-reference (to ICD-10) is perfectly equivalent or not [29].

We then reviewed ICD-10-AM codes that were not matched to an ORPHAcode and included ICD-10-AM codes that clearly and unambiguously referenced a RD, assigning a specific or broad ORPHAcode. Where possible, ORPHAcode assignment was performed according to Orphanet coding and linearisation rules, noting these rules typically apply to the application of ICD-10 codes to existing ORPHAcodes [30–32]. When manually applying an ORPHAcode for unmatched ICD-10-AM codes, and where a specific disorder code could not be found, the highest appropriate group or clinical code was applied, according to Orphanet classification procedure where possible [32]. Similarly, given the multisystemic nature of many RDs, a condition may belong to one or more classification groups. However, just a single group code was chosen for the purposes of this study, according to Orphanet linearisation rules where possible [31]. The subsequent list of matched ICD-10-AM codes was then reviewed based on their ICD-10-AM chapter (three-character) code; this was done to review certain higher level chapter codes for inclusion, due to potential issues with formatting and data entry within the source administrative data. We included three-character ICD-10-AM codes if all the four-character code subclassifications referenced a RD, or were very likely to reference a RD. A manual review of all proposed ICD-10-AM codes and their ORPHAcode match was then performed to help ensure validity. The parameters used for this manual review are outlined with examples in Additional file 5—Manual Review Parameters.

The final step in the development of the expanded Tasmanian Resource Set involved re-performing a period prevalence analysis to show if there were any diseases (identified by ICD-10-AM codes) with period prevalence in the Tasmanian population of > 1:2000. For period prevalence calculations, the number of people who were admitted or presented to hospital for each RD (ICD-10-AM code) during the study period was divided by the Tasmanian population size at the midpoint of the study period (December 2013) [21]. ICD-10-AM codes with a period prevalence of > 1:2000 were reviewed for suitability of inclusion as a RD. This expanded resource set of 1940 ICD-10-AM codes is provided in Additional file 6—Tasmanian Rare Diseases Resource Set.

### Assessment of resource use

A comparison of the resource use for patients with RDs and all patients using Tasmanian hospitals was separately examined using data held by the Tasmanian Department of Health for the period January 2018 until December 2022. This was done to enable a comparison to total statewide hospital activity and costs, and to examine the performance of the expanded Tasmanian Resource Set. Assessment of the admissions, LOS and costs for RD patients for RD and non-RD-related admissions relative to all public hospital inpatient activity data was analysed. A RD-related admission was one that included a RD code in any diagnosis field for that admission.

For the year 01 January 2020 to 31 December 2020, the Stringent Resource Set (1028 ICD-10-AM codes) and the Tasmanian Resource Set (1940 ICD-10-AM codes) were used to compare hospital admission costs for individuals with a RD-related admission in that year to all hospital admission costs in Tasmania for that year.

To identify RD patients within the Department of Health data, the two RDs resource sets, the Stringent Resource Set of 1028 ICD-10-AM codes and the Tasmanian Resource Set of 1940 ICD-10-AM codes, were provided to the data custodian within the Department of Health by the research team. Patients were identified by having one or more of the ICD-10-AM codes from the resource sets recorded in any diagnosis field within the period 1 January 2018 to 31 December 2022 in the public hospital inpatient dataset. Data for extraction included de-identified individual costs and specific disease costs.

Table 2 in Additional file 2 lists the data fields, derived fields and aggregate results provided by Department of Health for the study. No identifiable information was shared with the research team by the primary data custodian within the Department of Health, and the project did not require the data custodian (Department of Health) to access any additional individual records or collect any further information than that already held in the source dataset. Department of Health data were compared at an aggregate level with the TDLU linked data to validate RD prevalence findings.

**Table 2:** Comparison of hospital admissions and emergency department presentations between the RD and control cohorts in the linked dataset

	Number of individuals	Total hospital admissions (% RD-related)	Total hospital admissions per patient	Median number of admissions (mean)	Mean LOS (RD-related admission)	Total emergency department presentations (per patient)	Median emergency department presentations (mean)
RD cohort	13,806	177,655 (30.6%)	12.87	6.0 (13.2 ± 44.45)	3.3 (5.0)	95,056 (6.89)	4.0 (7.7 ± 10.93)
Control cohort	12,292	80,605 (0%)	6.56	3.0 (6.6 ± 27.6)	3.5	63,051 (5.13)	4.0 (5.8 ± 7.35)

Per patient episode costs were analysed from acute patient episodes, provided by the Department of Health data custodian from a derived table from the Department of Health patient administration system (iPM), which includes both principal Australian Refined Diagnosis Related Group (AR-DRG) (Diagnosis) codes and additional diagnosis codes based on ICD-10-AM coding. Costs per episode of care were calculated based on the primary AR-DRG (Diagnosis) code, with the cost for each AR-DRG based on the average cost per weighted separation, including depreciation multiplied by the Tasmanian acute separation cost. AR-DRG is an Australian admitted patient classification system, which provides a clinically meaningful way of relating the number and type of patients treated in a hospital to the resources required to treat them [33]. Each AR-DRG represents a class of patients with similar clinical conditions requiring similar hospital services [33]. Statistical readmissions, defined as a separation and readmission under a different care type, were included, as each episode is distinctly coded and funded. The cost weights for each AR-DRG were based on the National Acute cost weights for AR-DRG Version 10 rounds 23 and 24, and Version 11 weights for 2021–2022 [33]. Costs were deflated to 2021–22 Australian dollars using the price index for Government Final Consumption Expenditure for Hospitals and Nursing Homes [34].

## Results

### Initial cohort (case–control) analysis–stringent rare diseases resource set

Using the Stringent Resource Set of 1028 ICD-10-AM codes, there were 13,806 patients with at least one RD hospital admission, Emergency Department presentation or whose cause of death was due to a RD, identified in the linked datasets over the 14-year study period (between 01 January 2007 and 31 December 2020). In this RD cohort, 2119 patients (15.3%) had multiple (two to 11) distinctly different RD codes within their hospital records, although most patients had just one RD code (10,969; 79.5%). 13,502 patients (97.8%) in the RD cohort had 177,655 hospital admissions, and the median number of hospital admissions per patient over the study period was 6.0 (mean 13.2, ± 44.45). In the RD cohort, 54,331 (30.6%) of all hospital admissions were RD-related, and the mean LOS for RD-related hospital admissions was 5.0 days, compared to 3.3 days for non-RD-related admissions. 12,371 patients (89.6%) in the RD cohort had 95,056 Emergency Department presentations, and the median number of Emergency Department presentations per patient over the study period was 4.0 (mean 7.7, ± 10.93); however, just 1.1% (1047) of the Emergency Department presentations were coded as RD-related. During the study period, 5151 (37.3%) people in the RD cohort died, with a median age at death of 72 years; the cause of death was RD-related in half of the deaths (2589, 50.3%). There were 8655 patients alive at the end of the study period in the RD cohort, representing 1.5% of the Tasmanian population in December 2020.

Table 1 compares RD cohort and control cohort characteristics. In the control cohort of 12,545 patients matched by age, sex and geographical location, 12,292 patients (98.0%) had 80,605 hospital admissions during the study period, and the median number of hospital admissions per patient was 3.0 (mean 6.6, ± 27.6). 10,919 patients (87.0%) in the control cohort had 63,051 Emergency Department presentations, and the median number of Emergency Department presentations per patient over the study period was 4.0 (mean 5.8, ± 7.4). 2370 people in the control cohort died during the study period, with a median age at death of 77 years. Table 2 compares hospital admissions and Emergency Department presentations between the RD cohort and the control cohort. Table 3 compares the ICD-10-AM codes by ICD-10-AM Chapter in the 177,655 hospital admissions of the RD cohort and the 80,605 hospital admissions of the control cohort.

**Table 3:** Comparison of frequency ICD-10-AM codes by chapter in hospital admissions between the RD and control cohorts in the linked dataset

ICD-10-AM chapter	RD cohort		Control cohort	
	n ICD-10-AM code	%	n ICD-10-AM code	%
A00–B99 Certain infectious and parasitic diseases	22,793	3.4	7603	2.5
C00–D48 Neoplasms	56,880	8.4	15,005	5.0
D50–D89 Diseases of the blood and blood-forming organs and certain disorders involving the immune mechanism	24,766	3.7	3774	1.3
E00–E89 Endocrine, nutritional and metabolic diseases	57,077	8.5	22,899	7.6
F00–F99 Mental and behavioural disorders	15,333	2.3	10,295	3.4
G00–G99 Diseases of the nervous system	18,572	2.8	5236	1.7
H00–H59 Diseases of the eye and adnexa	4485	0.7	2718	0.9
H60–H95 Diseases of the ear and mastoid process	1678	0.2	617	0.2
I00–I99 Diseases of the circulatory system	39,063	5.8	21,743	7.2
J00–J99 Diseases of the respiratory system	22,652	3.4	9696	3.2
K00–K93 Diseases of the digestive system	34,480	5.1	17,352	5.8
L00–L99 Diseases of the skin and subcutaneous tissue	12,430	1.8	5030	1.7
M00–M99 Diseases of the musculoskeletal system and connective tissue	17,447	2.6	7816	2.6
N00–N99 Diseases of the genitourinary system	22,216	3.3	11,085	3.7
O00–O99 Pregnancy, childbirth and the puerperium	5205	0.8	4392	1.5
P00–P96 Certain conditions originating in the perinatal period	8196	1.2	1510	0.5
Q00–Q99 Congenital malformations, deformations and chromosomal abnormalities	8953	1.3	495	0.2
R00–R99 Symptoms, signs and abnormal clinical and laboratory findings, not elsewhere classified	54,573	8.1	24,037	8.0
S00–T98 Injury, poisoning and certain other consequences of external causes	21,264	3.1	13,743	4.6
U00–U49 Provisional assignment of new diseases of uncertain aetiology or emergency use	170	0.0	61	0.0
U50–U73 External causes of morbidity and mortality	8291	1.2	6223	2.1
U78–U88 Supplementary codes for chronic conditions	39,034	5.8	20,108	6.7
U90–U90 Healthcare associated infections	19	0.0	8	0.0
U91–U91 Syndrome, not elsewhere classified	23	0.0	2	0.0
U92–U92 Healthcare associated Staphylococcus aureus bloodstream infection [HA-SABSI]	5	0.0	0	0.0
V00–Y98 External causes of morbidity and mortality	51,634	7.6	25,925	8.6
Z00–Z99 Factors influencing health status and contact with health services	128,005	19.0	62,717	20.9

### Derivation of the expanded Tasmanian rare diseases resource set

**Fig. 1:**
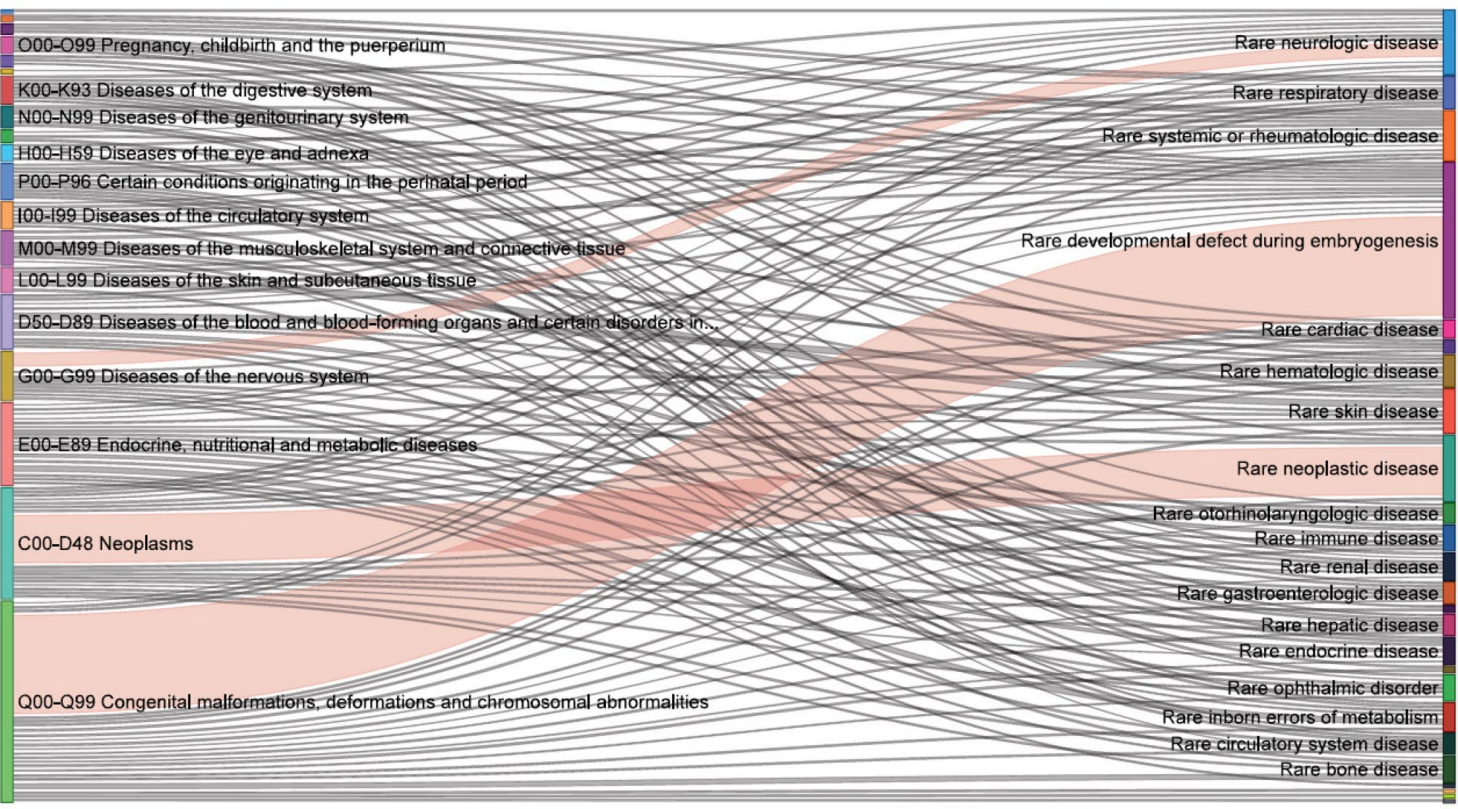
Count of ICD-10-AM codes from ICD-10AM chapters to Orphanet classification

Only 4% of patients from the validation cohort had one of the 1028 ICD-10-AM codes in any hospital admission or Emergency Department diagnosis field. Starting with a list of 16,464 ICD-10-AM codes that were linked to ICD-10 codes, 2591 ICD-10-AM codes were directly matched to an ICD-10 code that directly matched to an ORPHAcode. A further 2446 ICD-10-AM codes were included following manual review of unmatched ICD-10-AM codes, including previous version codes and supplementary codes for chronic conditions. These previously unmatched ICD-10-AM codes were manually assigned an ORPHAcode. This produced a list of over 4000 ICD-10-AM codes with one or more matching ORPHAcodes. The list was manually reviewed using the parameters outlined in Additional File 5—Manual Review Parameters, and over 2000 ICD-10-AM codes were subsequently removed. The final resource set contained 1940 ICD-10-AM codes cross-referenced to 1360 ICD-10 codes and 5823 ORPHAcodes (Additional File 6—Tasmanian Rare Diseases Resource Set). Table 4 shows the breakdown of ICD-10-AM codes in both resource sets by Chapter, and Table 5 shows the breakdown of linked ORPHAcodes by Orphanet classification for both resource sets. Figure 1 provides a visual flow of ICD-10-AM codes from ICD-10-AM Chapters to Orphanet classification for the Tasmanian Rare Diseases Resource Set.

**Table 4:** ICD-10-AM chapter breakdown for the stringent rare diseases resource set and the Tasmanian rare diseases resource set

ICD-10-AM chapter	Stringent RDs resource set (1028 codes)		Tasmanian RDs resource set (1940 codes)	
	n ICD-10-AM code	%	n ICD-10-AM code	%
A00–B99 Certain infectious and parasitic diseases	0	0.0	0	0.0
C00–D48 Neoplasms	98	9.5	405	20.9
D50–D89 Diseases of the blood and blood-forming organs and certain disorders involving the immune mechanism	67	6.5	119	6.1
E00–E89 Endocrine, nutritional and metabolic diseases	87	8.5	125	6.4
F00–F99 Mental and behavioural disorders	9	0.9	21	1.1
G00–G99 Diseases of the nervous system	45	4.4	127	6.5
H00–H59 Diseases of the eye and adnexa	4	0.4	23	1.2
H60–H95 Diseases of the ear and mastoid process	1	0.1	8	0.4
I00–I99 Diseases of the circulatory system	17	1.7	35	1.8
J00–J99 Diseases of the respiratory system	12	1.2	14	0.7
K00–K93 Diseases of the digestive system	7	0.7	13	0.7
L00–L99 Diseases of the skin and subcutaneous tissue	23	2.2	65	3.4
M00–M99 Diseases of the musculoskeletal system and connective tissue	187	18.2	59	3.0
N00–N99 Diseases of the genitourinary system	3	0.3	19	1.0
O00–O99 Pregnancy, childbirth and the puerperium	5	0.5	6	0.3
P00–P96 Certain conditions originating in the perinatal period	3	0.3	26	1.3
Q00–Q99 Congenital malformations, deformations and chromosomal abnormalities	458	44.6	868	44.7
R00–R99 Symptoms, signs and abnormal clinical and laboratory findings, not elsewhere classified	0	0.0	0	0.0
S00–T98 Injury, poisoning and certain other consequences of external causes	2	0.2	1	0.1
U00–U49 Provisional assignment of new diseases of uncertain aetiology or emergency use	0	0.0	2	0.1
U78–U88 Supplementary codes for chronic conditions	0	0.0	4	0.2
V00–Y98 External causes of morbidity and mortality	0	0.0	0	0.0
Z00–Z99 Factors influencing health status and contact with health services	0	0.0	0	0.0

**Table 5:** Orphanet classification breakdown for the stringent rare diseases resource set and the Tasmanian rare diseases resource set

Orphanet classification	Stringent RDs resource set (1028 codes)		Tasmanian RDs resource set (1940 codes)	
	n ORPHAcode	%	n ORPHAcode	%
Rare developmental defect during embryogenesis	151	33.0	2075	35.6
Rare neurologic disease	51	11.2	1064	18.3
Rare inborn errors of metabolism	24	5.3	507	8.7
Rare neoplastic disease	35	7.7	343	5.9
Rare skin disease	34	7.4	338	5.8
Rare bone disease	18	3.9	324	5.6
Rare endocrine disease	13	2.8	213	3.7
Rare hematologic disease	32	7.0	199	3.4
Rare ophthalmic disorder	9	2.0	177	3.0
Rare immune disease	13	2.8	168	2.9
Rare systemic or rheumatologic disease	33	7.2	140	2.4
Rare renal disease	7	1.5	90	1.5
Rare otorhinolaryngologic disease	6	1.3	44	0.8
Rare gastroenterologic disease	4	0.9	25	0.4
Rare hepatic disease	7	1.5	24	0.4
Rare respiratory disease	7	1.5	24	0.4
Rare cardiac disease	4	0.9	19	0.3
Rare odontologic disease	2	0.4	19	0.3
Rare circulatory system disease	1	0.2	12	0.2
Rare urogenital disease	2	0.4	8	0.1
Rare gynaecologic or obstetric disease	3	0.7	5	0.1
Rare maxillo-facial surgical disease	0	0.0	3	0.1
Rare infectious disease	0	0.0	1	0.02
Rare surgical cardiac disease	0	0.0	1	0.02
Rare abdominal surgical disease	1	0.2	0	0.0

There were 338 ICD-10-AM codes from the Walker et al. resource that did not match to the alignment data from Orphanet. This was because some represented an overall group ICD-10 code and were not specific codes, and some were no longer associated with the ORPHAcode, as that code has been broken down further.

Following development of the Tasmanian Resource Set, 55 (55%) validation cohort patients were identified in the expanded RD cohort. 663 (64.5%) of the ICD-10-AM codes from the Stringent Resource Set were present in any diagnosis field, compared to 1324 (68.3%) from the Tasmanian Resource Set. The Tasmanian Resource Set included 1940 ICD-10-AM codes and is provided in the Additional File 6—Tasmanian Rare Diseases Resource Set.

Using the Stringent Resource Set, the point prevalence of RD in December 2020 was 1.5% (Table 1) and the estimated period prevalence of RD, using the study period midpoint Tasmanian population (December 2013) of 513,015 [21], was 3.5%.

### Comparison of hospital resource use based on the stringent and Tasmanian rare diseases resource sets

Using the Stringent Resource Set (1028 ICD-10-AM codes), the number of people admitted in 2020 with a RD admission in the Department of Health dataset was 1822, compared with 3384 when using the Tasmanian Resource Set (Table 6). The total number of RD hospital admissions equated to 3.3% and 5.6% of statewide hospital admissions respectively. Based on the Tasmanian Resource Set, patients with a RD had a mean cost per hospital admission of AUD$11,310, compared to the statewide mean cost per hospital admission of AUD$6475. RD-related hospital admissions accounted for 9.1% of the total cost of Tasmanian public hospital admissions of AUD$979 million in 2020 (Table 6).

**Table 6:** Comparison to total Tasmanian hospital activity in 2020 for RD admissions in 2020, examined using data held by the Tasmanian department of health

2020 Tasmanian (hospital activity) admission data	People (% of total persons admitted)	Admissions (% of total admissions)	Mean admissions per patient	Mean LOS (in days)	Mean total days in hospital	Mean cost per admission	Total costs (AUD) for RD-related admissions (% of total statewide admissions cost)	Mean cost (AUD) per person
RD admissions identified using stringent rare diseases resource set	1822 (2.7%)	5050 (3.3%)	2.8	4.7	13.1	$12,343	$62 M (6.3%)	$34,211
RD admissions identified using Tasmanian rare diseases resource set	3384 (5.0%)	8526 (5.6%)	2.5	4.4	11.2	$11,310	$89 M (9.1%)	$28,497
Statewide total	68,133	151,196	2.2	3.1	7.0	$6475	$979 M	$14,370

**Fig. 2:**
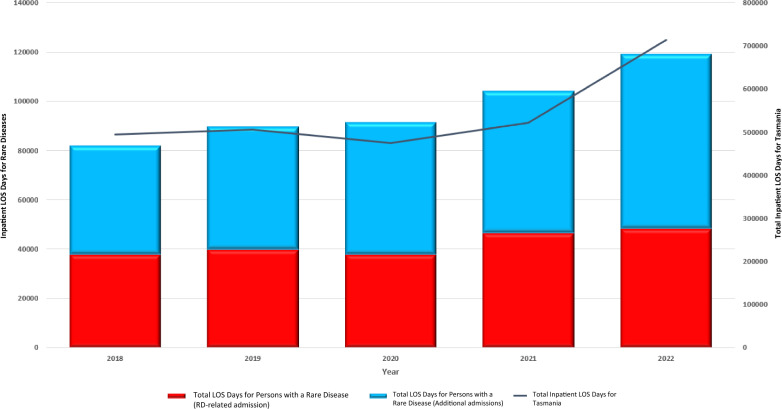
Total inpatient days for patients with a RD in Tasmanian Public Hospitals for RD and non-RD admissions, by Year, 2018–2022 (2021–22 AUD)

**Fig. 3:**
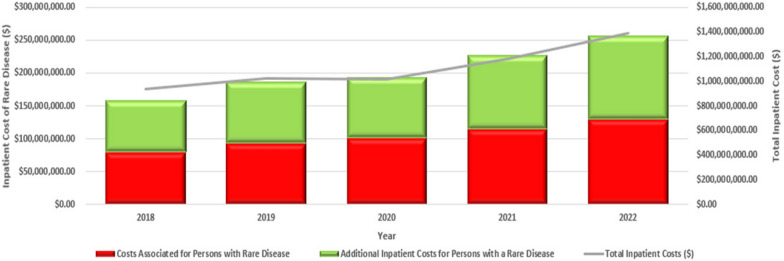
Total inpatient costs for patients with a RD in Tasmanian Public Hospitals for RD and non-RD admissions, and the Total Costs of Inpatient Admissions by Year, 2018–2022 (2021–22 AUD)

Trends in total inpatient days and costs were then analysed over five years from January 2018 to December 2022 for patients identified with a RD admission between 2018 to 2022 using the Tasmanian Resource Set. The total inpatient days for patients with a RD increased by 31% from 2018 to 2022, while the percent of total statewide inpatient days for patients with a RD remained stable: 16.6% in 2018 and 16.7% in 2022 (Fig. 2). The total inpatient costs for patients with a RD increased by 38.6% in real terms from 2018 to 2022, while the percent of total statewide hospital costs for patients with a RD increased from 16.8% in 2018 to 18.5% in 2022 (Fig. 3).

## Discussion

Using the Stringent Resource Set, the Tasmanian point prevalence in December 2020 was estimated to be 1.5%, which is comparable to previous studies that have used ICD-10 coding where point prevalence estimates have ranged from 1 to 2% [4, 14, 16]. However, quantifying RDs in healthcare systems that use ICD-10 coding is known to be challenging and is often thought to lead to undercounting and, thus, underestimation of their impact [35]. Poor ascertainment of RDs was confirmed during our study when only 4% from the validation cohort were identified using the Stringent Resource Set. Meanwhile, conservative estimates of RD point prevalence are 3.5–5.9% based on epidemiological data curated from literature and registries in the Orphanet database [7]. We subsequently started the process of developing an updated ICD-10-AM RDs resource set to improve RD identification, noting this resource is likely to remain useful for the foreseeable future given it is unclear when Tasmania or other Australian jurisdictions may adopt ICD-11 or ORPHAcodes. We note there is the intention to have one-to-one mapping between an ORPHAcode and an ICD-11 concept [30]. One particular issue for jurisdictions that use ICD-10-AM coding is that Orphanet alignment data [25] does not directly address ICD-10 to ICD-10-AM disambiguation issues, and there were several seemingly appropriate ICD-10-AM codes that were unrepresented in the Orphanet alignment data. While these were largely unrepresented due to differences between ICD-10 and ICD-10-AM coding, there were also several RD entities that were yet to be assigned an ORPHAcode. When performing this study, it was noted that ICD-10-AM appeared to have more specificity for certain RDs, such as several inborn errors of metabolism and rare congenital anomalies, compared to ICD-10. Further, it was noted that the presence of the fourth number in ICD-10-AM was useful in differentiating RD causes within a broader ICD-10 code that might not have been included in Orphanet alignment data. Using strict exclusion parameters, we included any unmatched ICD-10-AM codes that were very likely to represent RDs, and this process resulted in the expanded Tasmanian Resource Set having almost twice as many ICD-10-AM codes. This resource set consequently performed much better and was able to identify 55% of patients from the validation cohort. We then compared the performance of the Tasmanian Resource Set using public hospital admission data held by the Tasmanian Department of Health. The Tasmanian Resource Set identified approximately 86% more patients in 2020 compared to the Stringent Resource Set (3384 versus 1822 patients; 5.0% and 2.7% of all people admitted respectively). During that comparison, it was also noted that more patients were identifiable in the Department of Health data compared to the linked cohort using the Stringent Resource Set, as only 1599 RD patients in the linked cohort had an admission in 2020. The exact reasons for this difference were not fully ascertained, although it is possible the TDLU linked dataset was not as comprehensive as the full dataset used by the Department of Health. Work to understand this further, in parallel with a second round of linkage using the Tasmanian Resource Set, is underway.

Despite likely suboptimal ascertainment, the hospital impact of RDs in Tasmania was still found to be significant, consistent with other studies [4, 14, 16]. There were almost twice as many hospital admissions per patient for the RD cohort, and the median number of hospital admissions was also double. However, only 30.6% of hospital admissions for the RD cohort were coded as RD-related. As most RDs are chronic conditions, a significant proportion of these extra admissions for the RD cohort could be due to poor coding or a delayed RD diagnosis. The median LOS associated with RD-related hospital admissions was significantly higher compared to the control cohort, while the LOS for non-RD-related hospital admissions for the RD cohort was similar to the control cohort. Just 1.1% of Emergency Department presentations were coded as being RD-related. However, there were 34% more Emergency Department presentations per patient in the RD cohort, although the median number of presentations were approximately the same between cohorts. There were twice as many deaths across the study period in the RD cohort compared to the control cohort, with a RD coded as a cause of death in over half of all deaths in the RD cohort.

According to Department of Health public hospitals admissions data, the RD cohort also demonstrated a significant and disproportionate impact on healthcare resources in Tasmania as measured by higher cost per hospital admission and 49.6% higher mean cost per person. In 2020, using the Tasmanian Resource Set, individuals that had at least one RD hospital admission accounted for 5.0% of all hospital patients and 5.6% of all hospital admissions, with longer mean LOS than the general population. The total cost for RD-related hospital admissions in 2020 amounted to 9.1% of the state’s hospital admission costs, with the mean costs per RD patient twice that of the general population (Table 6). Importantly, when all admissions were included for RD patients, ascertained by having at least one RD admission between 2018 and 2022, each year the total hospital costs for these RD patients were estimated to account for around 20% of all hospital admission costs in Tasmania, and they appeared to moderately increase over time. For example, hospital costs for RD patients in 2020 were estimated to be AUD$193 million, which was approximately 19.0% of the total hospital costs in Tasmania in 2020. Our estimates support RD patients as having the highest spend for any group of admitted patients in the Tasmanian public hospital system; around 50% higher than the next disease group. Based on the Australian Burden of Disease Study (ABDS) condition list [36], which contains over 200 conditions in 17 groups, not including RDs, the disease group with the highest spending for admitted patients in public hospitals in Tasmania in 2020–21 was cardiovascular diseases, which accounted for AUD$133 million (11.2%) of spending [37].

Our findings are consistent with studies from other jurisdictions. Hospital service utilisation and costs associated with RD patients in WA in 2017 represented 4.6% of all hospital patients and 9.9% of all hospital discharges, with longer average hospital stays than the general population [4]. The total cost of hospital discharges for a RD cohort in WA amounted to 10.5% of the state’s inpatient hospital costs in 2010, noting their RD cohort was ascertained using a resource set of 1084 ICD-10-AM codes [4]. Internationally, a recent study in the United States has shown that RDs are associated with substantially higher health-care utilisation and costs compared to common conditions, with the total economic burden of RDs in the United States in 2019 estimated to be nearly USD$1 trillion; direct medical costs comprised nearly half of this burden, and higher hospital costs were associated with longer LOS and higher charges per discharge [17, 38]. Other jurisdictions, including Hong Kong [14], Ireland [15] and Italy [16], have also confirmed significant hospital service utilisation and costs for RD patients.

The results of our study suggest that reducing LOS for RD-related hospital admissions and reducing the total number of admissions for RD patients may significantly reduce costs. Meanwhile, including non-RD-related admissions for RD patients approximately doubles the total estimated hospital costs in each year. As most RDs are chronic conditions, further study will be undertaken to determine if a significant proportion of the additional hospital admissions for RD patients was due to poor coding, delayed RD diagnosis or other factors. Similarly, investigation into an apparent general increase in total inpatient LOS across Tasmania may be warranted, as RD alone is unlikely to explain the general increase in LOS that was observed.

Any strategy aimed at reducing hospital service utilisation and costs would need to align with patient and community expectations and lead to better care. This may include strategies to improve care pathways, reduce death rates through better treatment options, develop home and community-based cancer care, and establish patient communities and support. While the targetable factors underlying the significant hospital impact of RDs in Tasmania are yet to be determined, one likely beneficial strategy would be to identify and diagnose RD patients sooner [35, 39, 40].

## Limitations

We acknowledge several limitations in our study. First, the analysis did not distinguish rare diseases by severity, which is essential for understanding their varied impacts on the healthcare system. Second, the study did not differentiate between rare diseases that have treatments and those that do not, an important factor for guiding public health policies. Third, the data was limited to public hospitals, potentially omitting a significant portion of the population who receive care in private facilities. And fourth, the study did not separate the analysis of rare diseases between children and adults, which is crucial for understanding the specific needs and impacts on different age groups. In response to these limitations, we plan further analyses, incorporating data from both public and private hospitals to offer a complete picture of the impact of rare diseases across Tasmania. Future studies will employ the validated Tasmanian Rare Diseases Resource Set to analyse rare diseases by age, severity, and treatability, including a focus on childhood dementia and other severe conditions. Additionally, we will explore the differential impact of rare diseases that are treatable versus those that are not and compare early diagnosis outcomes.

Another limitation of this study is that our expanded Tasmanian Resource Set has not yet been validated by other groups. We included unmatched ICD-10-AM codes that were very likely to represent RDs, employed strict exclusion parameters, and prioritised exclusion over inclusion. This approach could have led to underestimation and, accordingly, further refinement of the Tasmanian Resource Set may be required. It is possible that ICD-10-AM may perform slightly better than ICD-10 in measures of RD specificity, but this is also yet to be systematically explored. It is worth noting that specificity was a common reason for exclusion in the Tasmanian Resource Set; Orphanet uses the best ICD-10 code available for some of their ORPHAcode associations, but that ICD-10 code may not be specific enough for RDs. Another limitation is that Orphanet nomenclature of RDs, being of European origin, is likely to show subtle variation when applied to Australian datasets, and that the Orphanet approach to cancer entities differs from the standard ICD-10 coding system. While Orphanet primarily focuses on morphology, ICD-10 typically categorises based on behaviour (malignant, benign, uncertain, or unknown), followed by topography, and then morphology. The RARECAREnet list was utilised to help mitigate these challenges; however, the classification of a rare cancer as being "not rare in Europe" does not necessarily imply the same prevalence in Tasmania.

Using ICD-10-AM diagnostic codes for estimating disease prevalence in hospital datasets has limitations due to potential inaccuracies in coding, lack of granularity in code categories, changes in coding practices over time, and the potential to overlook important clinical details. Variations in coding practices across facilities may also introduce bias, although in Tasmania hospital admission coding is undertaken centrally. These limitations highlight the importance of cautious interpretation and the need for complementary data sources to enhance accuracy and reliability. To mitigate these potential biases, we conducted case–control analyses which help to balance out the differences in coding practices across different hospitals and healthcare settings. However, this approach may not control for all variations in coding practice. For future research, we plan to further refine our methodology by matching controls based on specific conditions, such as comparing rare neurological diseases to common neurological diseases. This approach may help to provide a more accurate assessment of the healthcare burden of rare diseases, ensuring that variations in coding practices are minimised. Of note, while registry-based approaches may be a better way to ascertain RD prevalence in a population, this is not always practical, and there are RD registry gaps in Australia [41].

Using AR-DRG codes for estimating hospital costs also has limitations because they primarily classify patients based on broad resource use rather than focusing on individual cost factors, potentially overlooking complexities. Inaccuracies may arise from coding errors or omissions, and non-clinical cost influencers, such as overhead expenses, may not be fully accounted for, impacting the accuracy of cost estimates. To interpret mean admission costs, it also is necessary to consider how chronic conditions are represented between cohorts, especially since most rare diseases (RDs) are chronic disorders. Table 3 shows the frequency of ICD-10-AM codes by Chapter in hospital admissions for both the control cohort and the RD-cohort. This shows that chronic conditions (specifically ICD Chapter U78-U88 Supplementary codes for chronic conditions) are represented in the control cohort. In July 2015, 29 supplementary codes for chronic conditions (U78–U88) were implemented in ICD-10-AM to assist monitoring chronic conditions using hospitalisation data in Australia. These codes represent a discrete list of clinically significant chronic conditions, which are part of the patient’s current health status on admission. These supplementary codes for chronic conditions (U78–U88) mostly represent non-rare chronic diseases, and no supplementary codes for chronic conditions were included in the stringent RD resource set used to create the RD-cohort. For the next phase of the study using the Tasmanian RD resource set, we aim to match controls based on condition, i.e. rare neuro disease vs common neuro disease to further demonstrate the impact of rare diseases on the health system. Additionally, to help understand the impact of chronicity, as there are six RD codes in ICD Chapter U78-U88 (Supplementary codes for chronic conditions) included in the Tasmanian RD resource set, we can also examine the relative impact of those six RDs to the other non-rare chronic conditions in that Chapter.

Finally, there were 1261 more patients in the linked RD cohort verses the control cohort due to an issue with ICD-10-AM filtering across the data sets. The ICD-10-AM codes were recorded sometimes in the datasets with the decimal place and sometimes without. The initial filtering to establish the cohort and matched controls used the decimal point format only and, hence, several RD-eligible records were excluded. While it was possible to correct for the decimal issue and include several more RD patients in the cohort, it was not possible to do that for the control cohort, as the matching process had been completed. There are plans for a second round of linkage using the expanded Tasmanian Resource Set with matched controls.

## Conclusion

This study has provided an estimate of the prevalence of RDs in Tasmania and their impact on the hospital system. The results are comparable to other studies conducted in different jurisdictions and demonstrate a disproportionately high share of hospital service use and costs for patients with RDs. As a disease group, RDs likely represent the highest spending for admitted patients in public hospitals in Tasmania; 50% higher than cardiovascular disease. While modifiable factors underlying hospitalisation impact of RDs are poorly characterised in Tasmania and require further study, it is likely that a targeted allocation of resources could yield substantial benefits. Similarly, while the Tasmanian Rare Diseases Resource Set requires validation, it opens avenues for additional research and appeared to improve RD identification in a hospital dataset that utilises ICD-10-AM. Further study using the Tasmanian Rare Diseases Resource Set is planned, including analyses based on severity and treatability, to better understand the significant impact of RDs on hospitalisation in Tasmania.

## Supplementary Information

Supplementary Material 1.

Supplementary Material 2.

Supplementary Material 3.

Supplementary Material 4.

Supplementary Material 5.

Supplementary Material 6.

## Data Availability

The data that support the findings of this study are not openly available due to reasons of sensitivity and are available from the corresponding author upon reasonable request. Data are located in controlled access data storage at the Tasmanian Health Service.

## Abbreviations

ABDS: Australian Burden of Disease Study
AR-DRGs: Australian Refined Diagnosis Related Groups
ICD-10: International Classification of Diseases, Tenth Revision
ICD-10-AM: International Statistical Classification of Disease and Related Health Problems, Tenth Revision, Australian Modification
ICD-11: International Classification of Diseases, Eleventh Revision
IHACPA: Independent Health and Aged Care Pricing Authority
LOS: Length of stay
PPID: Project Person Identifier
RD: Rare disease
TDLU: Tasmanian Data Linkage Unit
