# Supplementary material for: Measuring the impact of rare diseases in Tasmania, Australia
Source: Orphanet J Rare Dis. 2024 Oct 28;19:399. doi: 10.1186/s13023-024-03343-2 (PMC11514960; doi:10.1186/s13023-024-03343-2)
Supplement: Supplementary file 1 — Supplementary Material 1. [file 13023_2024_3343_MOESM1_ESM.docx]

Researchable dataset merged by PPID (represents an individual in the population).

Data custodians attach clinical data and strip identifiers leaving PPID as unique identifier. Researchers received separate datasets, one from each custodian.

TDLU provides data custodians the PPID and Person Identifiers to attach approved clinical data items.

**Tasmanian Public Hospital Admitted Patients**

01/01/2007-31/12/2020

**Case Selection: 1084 ICD-10-AM codes**

**Tasmanian Public Hospital Emergency Department Presentations**

01/01/2000-31/12/2020

**Case Selection: 1084 ICD-10-AM codes**

**Tasmanian Cause of Death**

01/01/2006-31/12/2019

**Case Selection: 1084 ICD-10 -AM codes**

TDLU matched cases to its Master Linkage Map (MLM). TDLU selected matched controls (1:1) based on age, gender and SA2. Cases and controls extracted from the datasets below.

Data custodians identifies and extracts the cohort & provides TDLU with linkage variables.

TDLU links cohort to the MLM to identify the study cohort records. Controls are also selected from the MLM.

Linkage variables (refer to Additional file 2 - Study variables)

TDLU creates PPID to cases and controls and sends file back to data custodians.

Tasmanian Emergency Department

(Public Hospital)

Coded Cause of Death / Fact of Death

Tasmanian Admitted Patient Episodes

(Public Hospital)

2000-2020

COD 2006-2019

FOD 2000-2020

2007-2020

Person identifiers

PPID

Case refinement by Tasmanian frequency (1028 ICD-10-AM codes).

Control Cohort

(n= 12,545)

*Researchers reviewed datasets to ensure 1028 ICD-10-AM codes were appropriately present (RD cohort) or absent (control cohort) in any diagnosis field. 1261 additional PPIDs identified for the RD cohort due to ICD-10-AM decimal filtering issue.*

RD Cohort

(n=13,806)

Provided to each data custodian.
