# Supplementary material for: Measuring the impact of rare diseases in Tasmania, Australia
Source: Orphanet J Rare Dis. 2024 Oct 28;19:399. doi: 10.1186/s13023-024-03343-2 (PMC11514960; doi:10.1186/s13023-024-03343-2)
Supplement: Supplementary file 2 — Supplementary Material 2. [file 13023_2024_3343_MOESM2_ESM.docx]

## Additional file 2. Study variables

Table 1. Data provided to research team from TDLU linkage and data custodians.

| **Field** | **Data** | **Source** |
| --- | --- | --- |
| Field 1 | Project Person Identifier (PPID) |  |
| Field 2 | Date of birth: Month and year - MMYYYY |  |
| Field 3 | Patient sex |  |
| Field 4 | SA2 of residence |  |
| Field 5 | Indigenous status |  |
| Field 6 | Diagnoses | From diagnosis fields in all available datasets |
| Field 7 | ORPHA code | Assigned by researchers from Field 6: Diagnoses - ICD-10 codes |
| Field 8 | Orphanet linearisation parent (medical speciality) | Assigned by researchers from Field 7: ORPHA code |
| Field 9 | Unique Admission ID | Tasmanian Public Hospital Admitted Patient Episodes |
| Field 10 | Admission date (DD/MM/YYYY) | Tasmanian Public Hospital Admitted Patient Episodes |
| Field 11 | Discharge date (DD/MM/YYYY) | Tasmanian Public Hospital Admitted Patient Episodes |
| Field 12 | Length of stay (LOS) | Tasmanian Public Hospital Admitted Patient Episodes |
| Field 13 | Length of stay in Intensive Care Unit | Tasmanian Public Hospital Admitted Patient Episodes |
| Field 14 | Total psychiatric care days | Tasmanian Public Hospital Admitted Patient Episodes |
| Field 15 | Episode Diagnostic Related Group (DRG) including descriptions | Tasmanian Public Hospital Admitted Patient Episodes |
| Field 16 | Procedures (Procedure Codes 1-50) | Tasmanian Public Hospital Admitted Patient Episodes |
| Field 17 | Primary diagnosis (Diagnosis Code 1) | Tasmanian Public Hospital Admitted Patient Episodes |
| Field 18 | Additional diagnoses (Diagnosis Codes 2-100) | Tasmanian Public Hospital Admitted Patient Episodes |
| Field 19 | Major Diagnostic Category (MDC) | Tasmanian Public Hospital Admitted Patient Episodes |
| Field 20 | Service Related Group (SRG) | Tasmanian Public Hospital Admitted Patient Episodes |
| Field 21 | Cost weight | Tasmanian Public Hospital Admitted Episodes |
| Field 22 | Unique Presentation ID | Tasmanian Public Hospital Emergency Department Presentations |
| Field 23 | Triage date | Tasmanian Public Hospital Emergency Department Presentations |
| Field 24 | Triage category | Tasmanian Public Hospital Emergency Department Presentations |
| Field 25 | Waiting time to service delivery | Tasmanian Public Hospital Emergency Department Presentations |
| Field 26 | Length of non-admitted patient Emergency Department service episode | Tasmanian Public Hospital Emergency Department Presentations |
| Field 27 | Service Request Destination (Referred to on departure) | Tasmanian Public Hospital Emergency Department Presentations |
| Field 28 | Principal diagnosis code | Tasmanian Public Hospital Emergency Department Presentations |
| Field 29 | Major diagnostic block | Tasmanian Public Hospital Emergency Department Presentations |
| Field 30 | Cost weight | Tasmanian Public Hospital Emergency Department Presentations |
| Field 31 | Fact of death (Y/N) | Assigned by data custodians and/or TDLU |
| Field 32 | Date of Death (DD/MM/YYYY) | Tasmanian Coded Cause of Death |
| Field 33 | Age at death | Tasmanian Coded Cause of Death |
| Field 34 | Underlying cause of death (ICD-10) | Tasmanian Coded Cause of Death |
| Field 35 | Contributing cause of death (ICD-10) | Tasmanian Coded Cause of Death |

Table 2. Data provided by Tasmanian Department of Health data custodian to research team.

| **Field** | **Data** | **Source** |
| --- | --- | --- |
| Field 1 | AdmissionDateTime | Inpatient – iPM |
| Field 2 | DischargeDateTime | Inpatient – iPM |
| Field 3 | AdmissionTypeDesc | Inpatient – iPM |
| Field 4 | LOS | Inpatient – iPM |
| Field 5 | AdmMonthNameShort | Inpatient – iPM |
| Field 6 | AdmYearNumber | Inpatient – iPM |
| Field 7 | AGEY | Inpatient – iPM |
| Field 8 | U-Codes | Inpatient – iPM |
| Field 9 | U-CodeDescriptions | Inpatient – iPM |
| Field 10 | Z-Codes | Inpatient – iPM |
| Field 11 | Z-CodeDescriptions | Inpatient – iPM |
| Field 12 | SupplementaryCodes(Yes/No) | Inpatient – iPM |
| Field 13 | InfluencingCodes(Yes/No) | Inpatient – iPM |
| Field 14 | PalliativeCode(Yes/No) | Inpatient – iPM |
| Field 15 | DRG | Inpatient – iPM |
| Field 16 | DRG_Description | Inpatient – iPM |
| Field 17 | Total Cost | Tas Acute Costs Round 23 (2018-19) |
| Field 18 | Direct Cost | Tas Acute Costs Round 23 (2018-19) |
| Field 19 | Overhead Cost | Tas Acute Costs Round 23 (2018-19) |
| Field 20 | DX1-DX50 (All diagnosis codes) | Inpatient - iPM |
| Field 21 | PROC1-PROC30 (All procedure codes) | Inpatient - iPM |
| Field 22 | AdmissionAge | ED – Trak-ED |
| Field 23 | cEmergPatientAge | ED – Trak-ED |
| Field 24 | AdmissionLocation | ED – Trak-ED |
| Field 25 | InitialTriage | ED – Trak-ED |
| Field 26 | IntitialTriageDesc | ED – Trak-ED |
| Field 27 | ED_LOS_Hrs | ED – Trak-ED |
| Field 28 | TriageDateTime | ED – Trak-ED |
| Field 29 | DEMPrimaryDiagnosis | ED – Trak-ED |
| Field 30 | DEMPrimaryDiagnosisDesc | ED – Trak-ED |
| Field 31 | DEMPrimaryProcedure | ED – Trak-ED |
| Field 32 | ArrivalMode | ED – Trak-ED |
| Field 33 | InjuryCause | ED – Trak-ED |
| Field 34 | DepartureDestination | ED – Trak-ED |
| Field 35 | TransferDestination | ED – Trak-ED |
| Field 36 | InpatientAdmissionDiagnosis | ED – Trak-ED |
| Field 37 | Longitude | Derived based on THCI |
| Field 38 | Latitude | Derived based on THCI |
| Field 39 | Total Cost | Australian Emergency Care Classification and costs. |
| Field 40 | Direct Cost | Australian Emergency Care Classification and costs. |
| Field 41 | Overhead Cost | Australian Emergency Care Classification and costs. |
