# Supplementary material for: Measuring the impact of rare diseases in Tasmania, Australia
Source: Orphanet J Rare Dis. 2024 Oct 28;19:399. doi: 10.1186/s13023-024-03343-2 (PMC11514960; doi:10.1186/s13023-024-03343-2)
Supplement: Supplementary file 3 — Supplementary Material 3. [file 13023_2024_3343_MOESM3_ESM.docx]

## Additional file 3. Diseases removed as considered not rare in Tasmania based on estimated period prevalence > 1:2000.

1. Familial isolated dilated cardiomyopathy (ORPHA:154)
2. Cranial neuralgia (ORPHA:221109)
3. Disorder of iron metabolism and transport (ORPHA:309842)
4. Pericarditis (ORPHA:58208)
5. Trigeminal neuralgia (ORPHA:221091)
6. Patent arterial duct (ORPHA:706)
7. Sarcoidosis (ORPHA:797)
8. Meniere disease (ORPHA:45360)
9. Secondary polycythemia (ORPHA:98428)
10. Radiation proctitis (ORPHA:70475)
11. Normal pressure hydrocephalus (ORPHA:314928)

Table 1. ORPHAcode and corresponding ICD-10-AM codes removed from Walker et al list.

| **ORPHAcode** | **Orphanet Text** | **ICD-10-AM code** | **ICD-10-AM Text** |
| --- | --- | --- | --- |
| 154 | Familial isolated dilated cardiomyopathy | I42.0 | Dilated cardiomyopathy |
| 221109 | Cranial neuralgia | G50 | Disorders of trigeminal nerve |
|  |  | G50.1 | Atypical facial pain |
|  |  | G50.8* | Other disorders of trigeminal nerve |
|  |  | G50.9 | Disorder trigeminal nerve unspecified |
|  |  | G51 | Facial nerve disorders |
|  |  | G51.1 | Geniculate ganglionitis |
|  |  | G51.8 | Other disorders of facial nerve |
|  |  | G51.9 | Disorder of facial nerve unspecified |
|  |  | G52 | Disorders of cranial nerves |
|  |  | G52.0 | Disorders of olfactory nerve |
|  |  | G52.1* | Disorders of glossopharyngeal nerve |
|  |  | G52.2 | Disorders of vagus nerve |
|  |  | G52.3 | Disorders of hypoglossal nerve |
|  |  | G52.7* | Disorders of multiple cranial nerves |
|  |  | G52.8 | Disorders of other specified cranial nerves |
|  |  | G52.9 | Cranial nerve disorder unspecified |
|  |  | G53 | Cranial nerve disorders in diseases classified elsewhere |
|  |  | G53.0 | Postzoster neuralgia |
|  |  | G53.1 | Multiple cranial nerve palsies in infectious and parasitic diseases classified elsewhere |
|  |  | G53.2 | Multiple cranial nerve palsies in sarcoidosis |
|  |  | G53.3 | Multiple cranial nerve palsies in neoplastic disease |
|  |  | G53.8 | Other cranial nerve disorders in other diseases classified elsewhere |
| 309842 | Disorder of iron metabolism and transport | E83.1 | Disorders of iron metabolism |
| 58208 | Pericarditis | I30 | Acute pericarditis |
|  |  | I30.0 | Acute nonspecific idiopathic pericarditis |
|  |  | I30.1 | Infective pericarditis |
|  |  | I30.8 | Other forms of acute pericarditis |
|  |  | I30.9 | Acute pericarditis unspecified |
|  |  | I31.0 | Chronic adhesive pericarditis |
|  |  | I31.1 | Chronic constrictive pericarditis |
| 221091 | Trigeminal neuralgia | G50.0 | Trigeminal neuralgia |
| 706 | Patent arterial duct | Q25.0 | Patent ductus arteriosus |
| 797* | Sarcoidosis | D86 | Sarcoidosis |
|  |  | D86.0* | Sarcoidosis of lung |
|  |  | D86.1* | Sarcoidosis of lymph nodes |
|  |  | D86.2* | Sarcoidosis of lung with sarcoidosis of lymph nodes |
|  |  | D86.3* | Sarcoidosis of skin |
|  |  | D86.8* | Sarcoidosis of other and combined sites |
|  |  | D86.9* | Sarcoidosis unspecified |
| 45360 | Meniere disease | H81.0 | Meniere's disease |
| 98428 | Secondary polycythemia | D75.1 | Secondary polycythaemia |
| 70475 | Radiation proctitis | K62.7 | Radiation proctitis |
| 314928 | Normal pressure hydrocephalus | G91.2 | Normal-pressure hydrocephalus |

* Re-included in Tasmanian Rare Diseases Resource Set following expanded review.
