# Supplementary material for: Measuring the impact of rare diseases in Tasmania, Australia
Source: Orphanet J Rare Dis. 2024 Oct 28;19:399. doi: 10.1186/s13023-024-03343-2 (PMC11514960; doi:10.1186/s13023-024-03343-2)
Supplement: Supplementary file 5 — Supplementary Material 5. [file 13023_2024_3343_MOESM5_ESM.docx]

## Additional file 5. Manual review parameters

The following parameters were applied when performing the manual review (with examples):

1. Rare infections were excluded.
2. Rare neoplastic diseases were included.
   1. Apart from those that are considered 'not rare in Europe'.
3. Rare congenital malformations unlikely to be associated with disease were excluded.
   1. Some rare congenital malformations are likely to result in morbidity, while others are not. If the congenital malformation would usually be expected to either result in morbidity or be associated with a syndrome diagnosis, then it was flagged for inclusion.
   2. For example:
      1. accessory nipple (trait) was excluded.
      2. limb reduction defect was included.
4. Rare signs and symptoms were excluded.
   1. Any code that appeared to reference an abnormal sign or symptom was excluded, unless it was pathognomonic for a rare disease, or likely to identify someone with a rare disease.
   2. For example:
      1. G81.9 – hemiplegia was excluded.
      2. Epilepsy codes that captured epilepsy as a symptom were excluded, unless it was 'intractable' epilepsy.
      3. Thoracic aortic aneurysm (unruptured) may be silent and not rare and was excluded, whereas ruptured thoracic aortic aneurysm or dissection is rare and associated with significant morbidity and was included.
5. Codes that lacked specificity for a rare disease were excluded.
   1. This includes coding for conditions whose causes may include a rare disease, but it is not the most common reason for the code to be used. This was a common reason for exclusion. Orphanet uses the best ICD-10 code available for some of their ORPHAcode associations, but that ICD-10 code may not be specific enough for RD.
   2. For example:
      1. U79.4 - Disorder of intellectual development; J93.1 – Other spontaneous pneumothorax; P29.1 - Neonatal cardiac dysrhythmia; I42.8 - Other cardiomyopathies.
6. Conditions that are unlikely to be rare, despite an ORPHAcode match, were excluded.
   1. Codes that were specific for a disease, although the disease was likely not rare despite an ORPHAcode match.
   2. Excluded rare cancers based on the Not Rare in Europe (NRE) list.
   3. Other examples:
      1. G35 – Multiple Sclerosis; E22.2 – SIADH.
7. Rare pathology or imaging findings were excluded.
   1. Codes that appeared to reference abnormal pathology or imaging findings without being pathognomonic for a rare disease.
   2. For example:
      1. Excluded: E83.4 – Disorder of Mg metabolism; N12 – Tubule-interstitial nephritis; N13.3 - Other and unspecified hydronephrosis.
      2. Included: Pathogenic chromosomal copy number variants.
8. Trauma, mechanism of injury, procedure related, drug related, and infection related codes were excluded.
